# Regional Differences in the Permeability Barrier of the Skin—Implications in Acantholytic Skin Diseases

**DOI:** 10.3390/ijms221910428

**Published:** 2021-09-27

**Authors:** Anikó Kapitány, Barbara Medgyesi, Adrienn Jenei, Orsolya Somogyi, Lilla Szabó, Krisztián Gáspár, Gábor Méhes, Zoltán Hendrik, Klaudia Dócs, Péter Szücs, Zsolt Dajnoki, Andrea Szegedi

**Affiliations:** 1 Division of Dermatological Allergology, Department of Dermatology, Faculty of Medicine, University of Debrecen, 4032 Debrecen, Hungary; kapitany.aniko@med.unideb.hu (A.K.); medgyesibarbi@gmail.com (B.M.); jenei.adrienn@med.unideb.hu (A.J.); orsolya0625@gmail.com (O.S.); lillyhfg@gmail.com (L.S.); gaspar.krisztian@med.unideb.hu (K.G.); dajnoki.zsolt@med.unideb.hu (Z.D.); 2 Department of Dermatology, Faculty of Medicine, University of Debrecen, 4032 Debrecen, Hungary; 3 Gyula Petrányi Doctoral School of Allergy and Clinical Immunology, University of Debrecen, 4032 Debrecen, Hungary; 4 Department of Pathology, Faculty of Medicine, University of Debrecen, 4032 Debrecen, Hungary; gabor.mehes@med.unideb.hu; 5 Department of Forensic Medicine, Faculty of Medicine, University of Debrecen, 4032 Debrecen, Hungary; dr.hendrik.zoltan@gmail.com; 6 Department of Anatomy, Histology and Embryology, Faculty of Medicine, University of Debrecen, 4032 Debrecen, Hungary; docs.klaudia@anat.med.unideb.hu (K.D.); szucs.peter@med.unideb.hu (P.S.)

**Keywords:** acantolytic diseases, barrier function, cornified envelope, Darier’s disease, desmosome, Hailey–Hailey disease, keratinocyte, tight junction

## Abstract

The chemical milieu, microbiota composition, and immune activity show prominent differences in distinct healthy skin areas. The objective of the current study was to compare the major permeability barrier components (stratum corneum and tight junction (TJ)), investigate the distribution of (corneo)desmosomes and TJs, and measure barrier function in healthy sebaceous gland-rich (SGR), apocrine gland-rich (AGR), and gland-poor (GP) skin regions. Molecules involved in cornified envelope (CE) formation, desquamation, and (corneo)desmosome and TJ organization were investigated at the mRNA and protein levels using qRT-PCR and immunohistochemistry. The distribution of junction structures was visualized using confocal microscopy. Transepidermal water loss (TEWL) functional measurements were also performed. CE intracellular structural components were similarly expressed in gland-rich (SGR and AGR) and GP areas. In contrast, significantly lower extracellular protein levels of (corneo)desmosomes (DSG1 and CDSN) and TJs (OCLN and CLDN1) were detected in SGR/AGR areas compared to GP areas. In parallel, kallikrein proteases were significantly higher in gland-rich regions. Moreover, gland-rich areas were characterized by prominently disorganized junction structures ((corneo)desmosomes and TJs) and significantly higher TEWL levels compared to GP skin, which exhibited a regular distribution of junction structures. According to our findings, the permeability barrier of our skin is not uniform. Gland-rich areas are characterized by weaker permeability barrier features compared with GP regions. These findings have important clinical relevance and may explain the preferred localization of acantholytic skin diseases on gland-rich skin regions (e.g., Pemphigus foliaceus, Darier’s disease, and Hailey–Hailey disease).

## 1. Introduction

The density and size of hair follicles and sebaceous, eccrine, and apocrine glands are different across skin areas, creating a diverse environment for microbiota [1]. In parallel with the physiological properties and chemical milieu [1], the microbiota composition also varies in topographically different skin areas [2]. The skin microbiota is continuously sensed by our skin, and modulates the secretion of various innate immune mediators by keratinocytes and sebocytes, including interleukin-1α, complement components, and antimicrobial peptides [3]. Thus, significantly different epidermal levels of antimicrobial peptides (AMPs) and chemokines and distinct numbers of dermal T cells and dendritic cells are observed in different skin regions [4,5,6]. In sebaceous (sebaceous gland-rich (SGR)) and moist (apocrine gland-rich (AGR)) skin regions, AMPs are 2- to 40-fold upregulated at the mRNA level and 4- to 9-fold increased at the protein level compared to dry (gland-poor (GP)) skin areas [4,5]. Thus, the antimicrobial barrier of the skin cannot be considered uniform.

The regulation and maintenance of the antimicrobial and permeability skin barriers are closely connected [7,8,9,10]. Based on these data, we aimed to investigate the two major permeability barrier elements, the stratum corneum (SC) and the tight junctions (TJ), at the mRNA and protein levels [11,12]. Among the SC components, molecules related to cornified envelope formation, corneocyte desquamation, intercellular lipid lamellae formation, and (corneo)desmosome organization were analyzed along with TJ molecules. In addition, transepidermal water loss (TEWL) functional measurements were performed and the distribution and spatial organization of (corneo)desmosomes and TJs were compared using confocal microscopy.

## 2. Results

### 2.1. Distinct Barrier Functions Characterize Different Skin Regions

**Figure 1:**
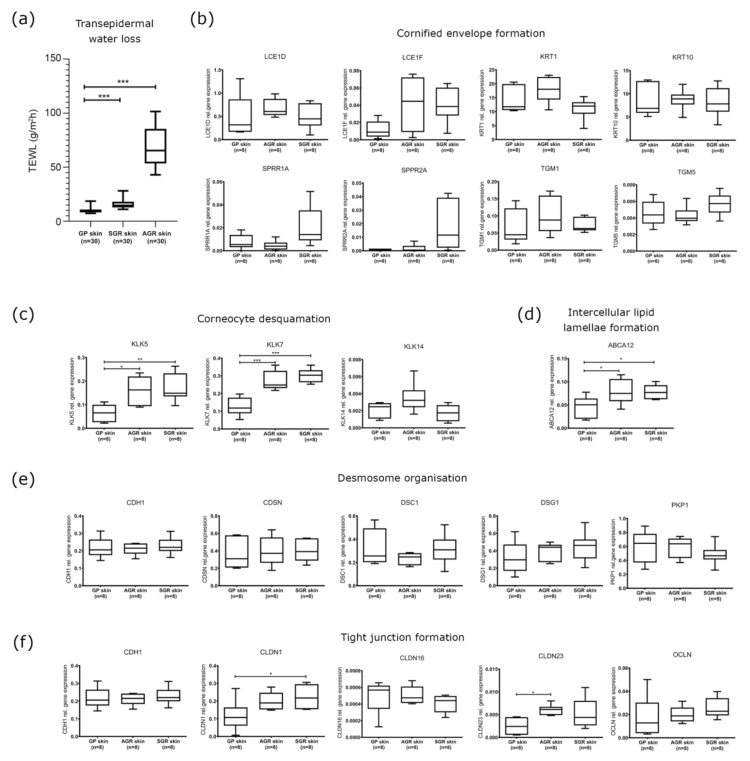
Different barrier functions and gene expression profiles of permeability barrier components characterize healthy GP, AGR, and SGR skin regions. (**a**) Transepidermal water loss levels in healthy GP, AGR, and SGR skin areas. Measurements were carried out with Tewameter TM300 (Courage and Khazaka, Cologne, Germany) on the flexural forearm, axilla, and forehead of healthy individuals (*n* = 30). Gene expression of the components of (**b**) cornified envelope formation, (**c**) corneocyte desquamation, (**d**) intercellular lipid lamellae, (**e**) desmosome organization, and (**f**) tight junction formation in healthy GP, AGR, and SGR skin regions were examined by qRT-PCR. The graphs show the mean ± 95% confidence interval of measured mRNA transcript levels (* *p* < 0.05, ** *p* < 0.01, *** *p* < 0.001, determined by one-way ANOVA followed by Sidac post hoc test). Abbreviations: ABCA12, ABC transporter 12B; AGR, apocrine gland-rich; CDH, cadherin; CDSN, corneodesmosin; CLDN, claudin; DSC, desmocollin; DSG, desmoglein; GP, gland-poor; KLK, kallikrein-related peptidase; KRT, keratin; LCE, late cornified envelope; OCLN, occludin; PKP, plakophilin; SGR, sebaceous gland-rich; SPRR, small proline-rich protein; TEWL, transepidermal water loss; TGM, transglutaminase.

SGR and AGR regions were characterized by significantly higher TEWL levels compared to GP areas, indicating differences in barrier function among healthy skin regions. Differences were more prominent between the AGR and GP areas (6.89 fold increase) than in SGR versus GP areas (1.57 fold increase) (Figure 1a).

### 2.2. Cornified Envelope Intracellular Structure Molecules Similarly Expressed in Distinct Skin Areas

**Figure 2:**
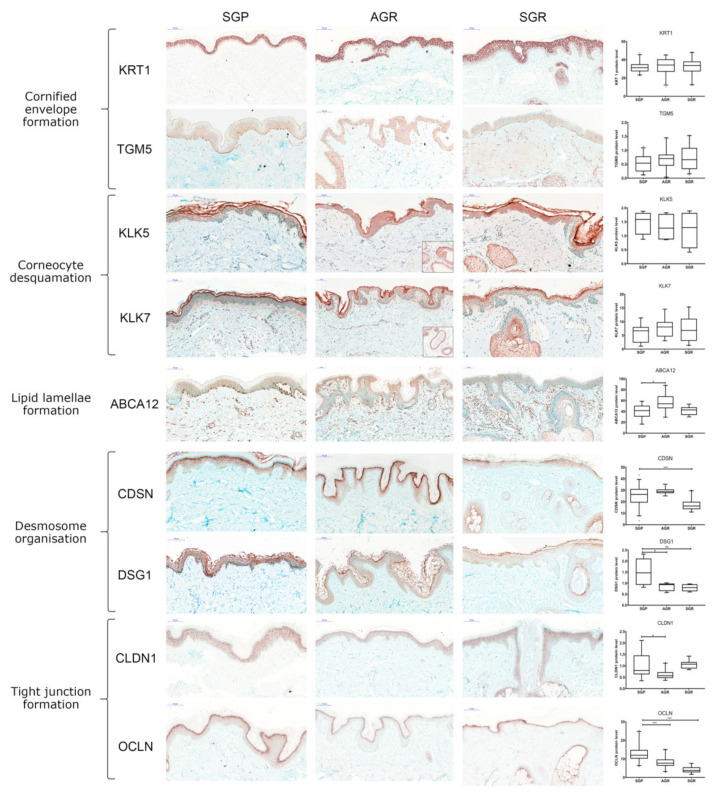
Prominently weakened cell junction components characterize SGR and AGR skin regions compared with GP areas under steady-state. Representative images for immunostaining and quantification of epidermal levels of KRT1, TGM5, CDSN, DSG1, CLDN1, OCLN, ABCA12, KLK5, and KLK7 in healthy GP, AGR, and SGR skin sections. Bar = 100 μm. Means ± 95% confidence intervals for protein levels are shown. (* *p* < 0.05, ** *p* < 0.01, *** *p* < 0.001, determined by one-way ANOVA followed by Sidac post hoc test) Abbreviations: ABCA12, ABC transporter 12B; AGR, apocrine gland-rich; CDSN, corneodesmosin; CLDN, claudin; DSG1, desmoglein 1; GP, gland-poor; KLK, kallikrein-related peptidase; KRT, keratin; OCLN, occludin; SGR, sebaceous gland-rich; TGM, transglutaminase.

No significant differences were detected among the three skin regions in the mRNA and protein expression of molecules taking part in cornified envelope formation, including KRT1, KRT10, LCE1D, LCE1F, SPRR1A, SPRR2A, TGM1, and TGM5 (Figure 1b and Figure 2).

### 2.3. Increased Expression of KLK Proteases and the ABCA12 Lipid Transporter Characterize SGR and AGR Skin Regions

KLK5 and KLK7 enzymes, which play crucial roles in corneocyte desquamation [13,14,15,16], were significantly upregulated at the mRNA level in SGR and AGR areas compared with GP regions (Figure 1c). Protein levels for KLK5 and KLK7 in the interfollicular and follicular epidermis were similar in the three sample groups. However, IHC revealed stronger positive staining for KLK5 and KLK7 in the apocrine and sebaceous glands of AGR and SGR regions, resulting in substantial differences between gland-rich and GP skin regions (Figure 2). The ABCA12 mRNA levels were significantly higher in both SGR and AGR regions versus GP areas (Figure 1d). Moreover, the ABCA12 transporter was slightly, but significantly, upregulated at the protein level in the AGR epidermis compared with GP skin.

### 2.4. Decreased Protein Expression of (Corneo)Desmosome and Tight Junction Components in SGR and AGR Skin Regions

Concerning the (corneo)desmosome components, the mRNA expression of CDH1, CDSN, DSC1, DSG1, and PKP1 did not differ significantly among the healthy skin areas (Figure 1e). At the protein level, DSG1 was significantly downregulated in both SGR and AGR skin, and CDSN was significantly decreased in SGR compared with GP skin (Figure 2).

Regarding the TJ components, at the mRNA level, CLDN1 was upregulated in AGR regions, and CLDN23 was upregulated in SGR regions compared to GP skin, while similar mRNA levels were detected for CLDN16, CDH1, and OCLN (Figure 1e). Protein expression of TJ molecules did not coincide with their gene expression. OCLN levels were significantly lower in SGR and AGR areas and CLDN1 protein was significantly downregulated in AGR skin compared to GP areas (Figure 2).

### 2.5. Distinct Organization of Cell Junctions in the Permeability Barrier of Different Regions

**Figure 3:**
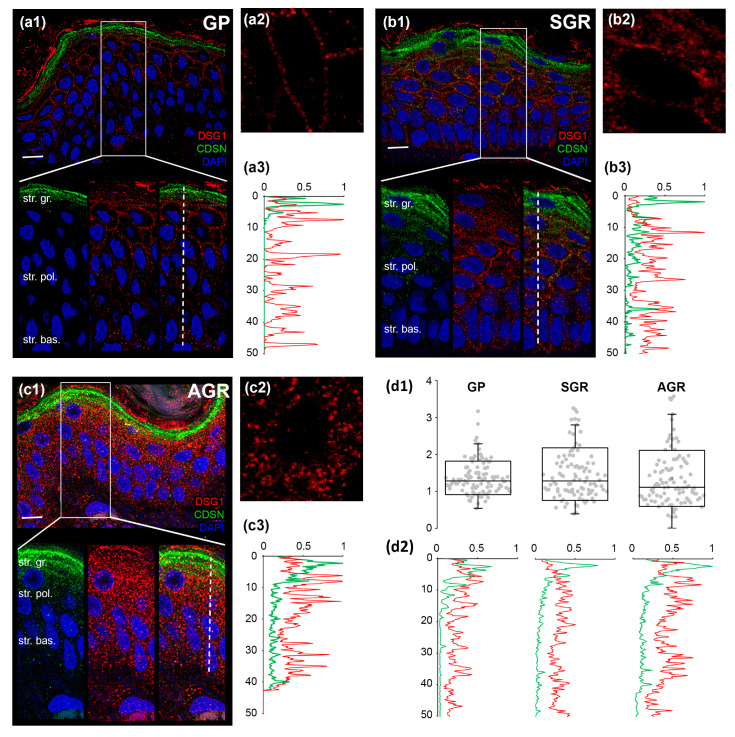
Less organized (corneo)desmosomes characterize SGR and AGR regions compared to GP skin areas. Double immunofluorescent staining against DSG1 (red) and CDSN (green) was performed to visualize their expression in the epidermis of dry (GP; (**a1**)), sebaceous (SGR; (**b1**)), and moist (AGR; (**c1**)) skin types. Nuclei were stained with DAPI (blue). Strong expression of CDSN was apparent in the stratum granulosum of the epidermis of all skin regions. In SGR and AGR areas, a prominent widening of the CDSN expression towards stratum spinosum was observed, as indicated by the elevated line of the individual (**b3**,**c3**) and normalized green curves in (**d2**). DSG1 staining was observed in deeper layers of the epidermis in SGR and AGR types (compare individual red curves in (**a3**,**b3**,**c3**) and the normalized red curves in (**d2**). DSG1 dots were more regularly spaced around keratinocytes of the GP epidermis (**a2**), whereas the DSG1 dots were bunched up and irregularly distributed in SGR type (**b2**) and, even more, in AGR type (**c2**) skins. This difference in the regularity was indicated by the smaller dispersion of data points in the GP skin region compared with the SGR and AGR areas in (**d1**). Each dot represents the distance between two adjacent tight junction structures around keratinocytes. Scale bar on all images: 10 µm. The *Y*-axis indicates the distance in micrometers in (**a3**,**b3**,**c3**,**d1**,**d2**). The *X*-axis in (**a3**,**b3**,**c3**,**d2**) shows the normalized intensity of the immunostaining.

Next, we determined whether the decreased protein expressions of (corneo)desmosome and TJ molecules in AGR and SGR areas were associated with the altered organization of these cell junction structures. DSG1 and CDSN, representing (corneo)desmosomes, were detected by double immunofluorescent staining (Figure 3(a1,b1,c1)). In GP areas, as expected, strong expression of CDSN was apparent in the stratum granulosum but not the deeper cell layers (Figure 3(a1,a3)). DSG1, unsurprisingly, was localized mostly in the stratum granulosum with decreasing levels towards the basal epidermal layer (Figure 3(a1,a3)). The two proteins showed strong staining and dense co-localization, representing (corneo)desmosome formation, in a narrow (1–2 cell) layer of stratum granulosum (Figure 3(a1,a3,d2)). To determine the level of (corneo)desmosome organization, we analyzed their regularity by measuring the distance between two adjacent immunostained DSG1 dots in a cross-section of the cell membrane in the stratum granulosum. DSG1 dots were regularly spaced around the keratinocytes in GP epidermis (Figure 3(a2,d1)).

In contrast to GP areas, widened CDSN expression towards the stratum spinosum was detected in SGR and AGR regions (Figure 3(b1,b3,c1,c3)). In addition, DSG1 expression in SGR and AGR areas was detected in both the stratum granulosum and the deeper layers (Figure 3(b1,b3,c1,c3)). Consequently, co-localization of CDSN and DSG1 was less dense and spread over a wider range of cell layers compared to the GP area (Figure 3(b1,b3,c1,c3,d2)). Moreover, the spatial distribution of (corneo)desmosomes, represented by DSG1 dots, was less organized, bunched up, and irregularly distributed in SGR and, even more, in AGR regions (Figure 3(b2,c2,d1)).

**Figure 4:**
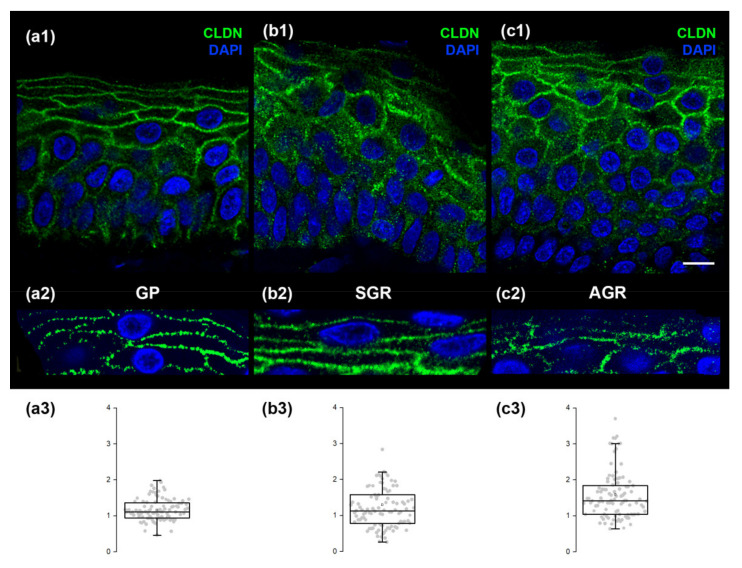
CLDN1, representing tight junctions, exhibits a highly regular pattern in GP regions but is less organized in SGR and AGR areas. Micrographs of single 1 μm-thick confocal optical sections demonstrate the distribution of CLDN1 through the layers of the epidermis. The strongest expression of CLDN1 was observed in the stratum granulosum of all skin regions (**a1**,**b1**,**c1**), while immunostaining widened towards the stratum spinosum of SGR and AGR skin areas (**b1**,**c1**). The distribution of CLDN1 immunostained dots was highly regular in GP skin (**a2**), but was less regularly organized in SGR (**b2**) and formed clusters in AGR (**c2**). Quantification of the differences in regularity is shown as box plots in (**a3**,**b3**,**c3**); each dot represents the distance between two adjacent desmosome structures around keratinocytes. Note the narrower dispersion of data points in (**a3**) compared with (**b3**,**c3**). Scale bar: 10 µm The *Y*-axis represents distances in micrometers in (**a3**,**b3**,**c3**).

Regarding CLDN1 (representing TJ structures) immunofluorescent staining in GP regions, the stratum granulosum exhibited the strongest expression (Figure 4(a1)). The distribution of CLDN1 dots was highly regular in GP skin regions (Figure 4(a2,a3)). In contrast, the immunostaining of CLDN1 widened towards the stratum spinosum (Figure 4(b1,c1)) in SGR and AGR regions. Moreover, immunostained dots of CLDN1 were irregularly distributed in SGR and, even more, in AGR skin in the granular layer (Figure 4(b2,b3,c2,c3)).

## 3. Discussion

The anatomy and physiology of our skin exhibit significant regional differences. The microbiota composition and immune tuning of our skin also exhibit prominent regional differences [2,4,5,6,17]. Thus, regional differences may also exist in the skin permeability barrier. We aimed to comprehensively analyze the permeability barrier of different healthy skin regions using functional measurements and mRNA- and protein-based methods.

According to our functional analysis, TEWL is significantly higher in SGR and AGR skin regions compared to GP areas, indicating that AGR and SGR regions may have weaker permeability barriers, although the extremely high numbers of sweat glands can also influence the TEWL in the axillar area. This finding is in good concordance with the results of Kleesz et al., which demonstrate higher TEWL in SGR and AGR skin compared to the GP region, with the highest TEWL in AGR areas [18].

During the molecular analysis of the intracellular structure proteins, no differences in intracellular structure proteins were detected between the three skin areas. Although similar mRNA levels for extracellular junctions were detected in the three skin areas (Figure 1), AGR and SGR areas expressed significantly decreased protein levels of several (corneo)desmosome and TJ molecules (Figure 2). Alterations occurred only in the expression of extracellular junctional components and only at the protein level, which suggests that extracellular proteolytic degradation, rather than decreased protein production, is responsible for the weakened permeability barrier in AGR and SGR skin areas. Supporting this idea, we detected prominent mRNA expression levels of the extracellular proteases KLK5 and KLK7 and the expression levels were significantly higher in AGR and SGR skin versus GP. Moreover, KLKs increased at the protein level in AGR and SGR regions due to their production by the apocrine and sebaceous glands, respectively. These findings are in line with a study demonstrating higher KLK levels in sweat collected from the face and axilla [19]; however, the direct proteolytic degradation of TJ molecules by KLKs needs to be confirmed in the future by zymographic analysis.

As DSG1, DSC1, and CDSN are the major cleavage targets of KLK5 and KLK7, these enzymes may be directly responsible for the more pronounced degradation of (corneo)desmosomes in SGR and AGR skin [14,20]. In addition, KLKs activate multiple enzymes involved in TJ degradation (e.g., PAR2, matrix metalloproteases) and may be at least partially responsible for TJ weakness in SGR and AGR regions [21,22,23]. Moreover, region-specific bacteria and fungi (Propionibacterium and Malassezia on SGR; Corynebacterium, Proteobacteria, and Staphylococcus aureus on AGR skin [2,17]) influence the permeability barrier by producing exogenous serine and cysteine peptidases [24,25,26,27,28,29,30].

The ABCA12 lipid transporter plays a crucial role in lipid lamellae formation, and its mRNA levels were highly upregulated in SGR and AGR regions. Moreover, significant increases were detected at the protein level in the AGR region compared with GP. These differences in ABCA12 expression highlight the heterogeneous nature of our skin; however, further investigation is needed.

To validate our molecular findings and understand the organization and distribution of (corneo)desmosomes and TJs in different skin areas, we used confocal microscopy. The (corneo)desmosomes and TJs in the GP region were regularly distributed, as expected. In contrast, AGR and SGR areas were characterized by prominently disorganized junction structures. These significant differences were found in healthy skin regions, highlighting the important clinical relevance of our findings.

**Figure 5:**
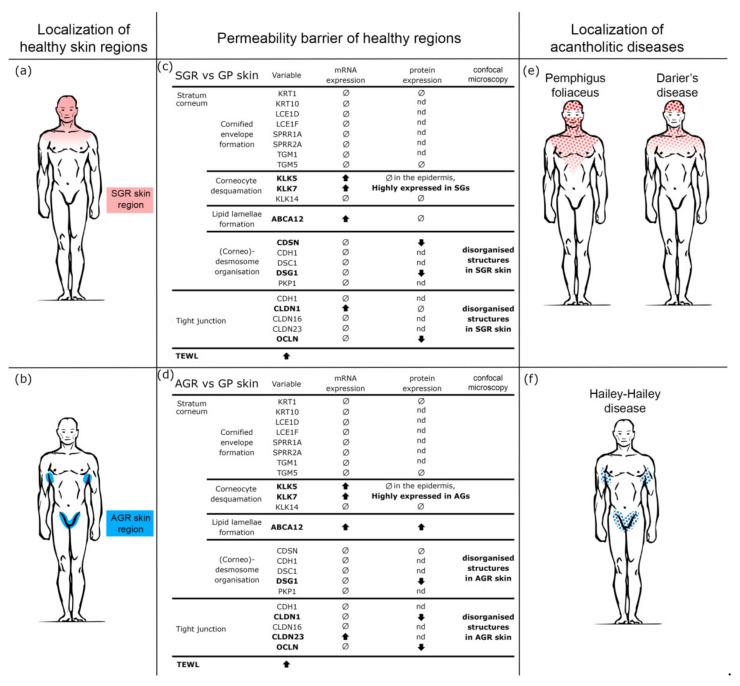
The weakened permeability barrier of SGR and AGR skin regions may explain the characteristic region preference of acantholytic skin diseases. (**a**,**b**) Based on the distinct chemical milieu, microbiota composition, and immune tuning, three topographically different healthy skin regions can be distinguished, including gland-poor (GP), apocrine gland-rich (AGR), and sebaceous gland-rich (SGR) skin areas. (**c**,**d**) According to our current findings, permeability barrier-related differences exist in these healthy skin regions. These differences primarily affect cell junctions; expression of protein components and cell membrane organization were significantly lower in SGR and AGR versus GP regions. These differences turned up at the protein rather than mRNA levels, indicating the role of enzymatic degradation in the weakened cell junctions in AGR and SGR areas. These results are in line with the strong KLK5 and KLK7 positivity in these areas. The weakened permeability barrier in AGR and SGR regions was confirmed by TEWL measurements. (**e**,**f**) The results of our study explain the well-known but unexplained clinical observations that some acantholytic skin disorders have preferred localization. Pemphigus foliaceus (PF) develops first in SGR, while Darier’s disease (DD) and Hailey–Hailey disease (HHD) mainly develop their main signs in SGR and AGR skin regions. Our present data indicate that these skin regions have weakened permeability barriers under steady-state and are the weak links of our skin.

In summary, healthy AGR and SGR skin regions are equipped with higher TEWL, higher levels of KLK enzymes, decreased expression of certain (corneo)desmosome and TJ proteins, and irregularly distributed (corneo)desmosomes and TJs compared to GP areas. These results indicate that AGR and SGR regions are the so-called weak links of our skin (Figure 5). From the clinical perspective, our present findings may explain the characteristic lifetime occurrence and topographical distribution for some acantholytic skin diseases, including pemphigus foliaceus (PF) [31,32,33], Darier’s disease (DD) [34,35], and Hailey–Hailey disease (HHD) [35,36]. The common feature of these skin disorders is the loss of keratinocyte adhesion due to the damaged desmosome/corneodesmosome formation caused by mutations in DD and HHD and by autoantibodies in PF [31,32,33,34,35,36,37].

In DD and HHD, mutations in the ATP2A2 and AT+-P2C1 Ca^2+^ pump genes cause damaged desmosome/(corneo)desmosome formation [32,33,37]; however, clinical signs usually appear after adolescence and nearly always in SGR/AGR areas despite being present throughout the lifetime and in all keratinocytes [32,36,37]. We hypothesize that genetic alterations of the desmosomes in DD and HHD lead to the manifestation of clinical signs after puberty and mainly in the SGR and AGR skin regions due to the enhanced KLK enzymes, degradation of junction proteins, and, consequently, weakened desmosomes and TJs in these areas develop only after adolescence. Thus, these specific regions manifest the mentioned genetic alterations. Both the characteristic SGR/AGR localization of these diseases (Figure 5) and their common appearance after puberty can be explained based on our results. In PF, although anti-desmoglein1 antibodies bind to desmosomes in the whole skin surface; however, the first and dominant clinical signs of bulla formation occur in SGR areas, where weaknesses in (corneo)desmosomes/TJs sensitize these regions to disease development.

Significant therapeutic consequences may also be drawn from these data. Modulation of those enzymes that are likely responsible for enhanced degradation of extracellular junction molecules and, consequently, the weakened cell junctions of SGR/AGR skin areas, by locally applied inhibitors or antibiotics, may delay or obstruct the occurrence of these diseases.

In conclusion, our skin is a topographically heterogeneous, non-unified organ represented not only by regionally distinct chemical and microbial milieu, and different immune tuning, but also by distinct permeability barrier characteristics. These distinct characteristics have important impacts on the pathogenesis, development, and localization of skin diseases. These data should be taken into consideration for both scientific and therapeutic approaches.

## 4. Materials and Methods

### 4.1. Measurement of Transepidermal Water Loss

Measurements were performed under standardized laboratory conditions at a temperature of 22–25 °C and a humidity level of 40–60% in healthy individuals (*n* = 30). Individuals were allowed to adapt to room conditions for at least 15 min before the measurements. The Tewameter TM300 (Courage and Khazaka, Cologne, Germany) was used to measure transepidermal water loss (TEWL) (g/hm^2^) on the flexural forearm (representing the GP region), axilla (representing the AGR area), and forehead (representing the SGR region) (Figure 5). Measurements were performed in triplicate for 30 s each.

### 4.2. Skin Biopsies

Skin punch biopsies (0.5–1 cm^2^) were taken from GP (shin, lower arm), SGR (face, behind the ear, hairy scalp), and AGR (axilla) skin sites (*n* = 8 per area) of healthy individuals (mean age ± standard deviance (SD): 56.25 ± 13.11) undergoing plastic surgery (Table 1). Written, informed consent, according to the Declaration of Helsinki principles, was obtained by all individuals before participating in the study. The study was approved by the local ethics committee of the University of Debrecen.

Skin samples were further defined histologically according to the number of sebaceous and apocrine glands in the field of view at 100× magnification, as previously described [4,6]. AGR samples were included in the study if at least 2 apocrine glands were observed, while SGR samples were used if at least 3 sebaceous glands were in the field of view. Samples were considered SGP if neither apocrine nor sebaceous gland were observed.

### 4.3. Processing of Skin Biopsy Specimens

All biopsies were cut into two pieces. For immunohistochemistry and immunofluorescent staining, samples were formalin-fixed and paraffin-embedded (FFPE). For quantitative RT-PCR, samples were stored in RNAlater (Qiagen, Hilden, Germany) at −70 °C until RNA isolation.

### 4.4. RNA Isolation, Reverse Transcription

Samples were homogenized in Tri reagent (Sigma-Aldrich, St. Louis, MO, USA) using a TissueLyser (Qiagen) with autoclaved metal beads (Qiagen). RNA concentration and purity were measured with a NanoDrop spectrophotometer (Thermo Fisher Scientific, Bioscience, Waltham, MA, USA), and RNA quality was checked using an Agilent 2100 Bioanalyzer (Agilent Technologies, Santa Clara, CA, USA). For RT-PCR, cDNA was synthesized from the isolated RNA using a High Capacity cDNA Archive Kit (Invitrogen, Life Technologies, Carlsbad, CA, USA).

### 4.5. Real-Time Polymerase Chain Reaction (RT-qPCR)

RT-PCR was performed in triplicate using pre-designed MGB assays (Thermo Fisher, Budapest, Hungary). Reactions were performed with an ABI PRISM^®^ 7000 Sequence Detection System. The following TaqMan Gene Expression assays were used: PPIA (Hs99999904_m1), ABCA12 (Hs00292421_m1), CDH1 (Hs01023895_m1), CDSN (Hs00169911_m1), CLDN1 (Hs00221623_m1), CLDN16 (Hs00198134_m1), CLDN23 (Hs01013638_s1), DSC1 (Hs00245189_m1), DSG1 (Hs00355084_m1), KLK14 (Hs00983577_m1), KLK5 (Hs01548153_m1), KLK7 (Hs00192503_m1), KRT1 (Hs00196158_m1), KRT10 (Hs00166289_m1), LCE1D (Hs04224967_gH), LCE1F (Hs00820275_sH), OCLN (Hs00170162_m1), PKP1 (Hs00240873_m1), PPIA (Hs99999904_m1), SPRR1A (Hs00954595_s1), SPRR2A (HS03046643_s1), TGM1 (Hs00165929_m1), TGM5 (Hs00909973_m1). Relative mRNA levels were calculated using the 2-ΔΔCt method normalized to the expression of PPIA mRNA.

### 4.6. Immunohistochemistry

For immunohistochemistry (IHC) analyses, paraffin-embedded sections were deparaffinized. Heat-induced antigen retrieval was performed, and sections were treated with H_2_O_2_ for 10 min. Sections were stained with primary antibodies against human ABCA12 (rabbit polyclonal IgG, GTX51202, GeneTex), human CDSN (rabbit polyclonal IgG, HPA054184, Sigma-Aldrich, St. Louis, MO, USA), human CLDN1 (rabbit polyclonal IgG, ab15098, Abcam, Cambridge, UK), human DSG1 (rabbit polyclonal IgG, NBP1-84567, Novus Biologicals, Centennial, CO, USA), human KLK5 (rabbit polyclonal IgG, ab7283, Abcam), human KRT1 (rabbit monoclonal IgG, ab185628, Abcam), human OCLN (rabbit monoclonal IgG, ab216327, Abcam), and human TGM5 (rabbit polyclonal IgG, ab133786, Abcam).

Subsequently, an anti-mouse/rabbit horseradish peroxidase-conjugated secondary antibody was employed (EnVision FLEX Mini Kit, High pH; Agilent, Dako, Santa Clara, CA, USA). Before and after incubating with antibodies, samples were washed 3 times for 5 min each. Staining was detected with the Vector NovaRed Kit (Vector Laboratories, Burlingame, CA, USA). Sections were counterstained with methylene green, dehydrated, and covered with a glass coverslip. Detection of each protein was carried out on all sections in parallel at the same time to evaluate comparable protein levels. Positive and negative controls were used to normalize staining against all proteins. The sections were digitized using Whole Slide Imaging technology. Pannoramic Viewer (Budapest, Hungary) was used to evaluate the degree of staining.

### 4.7. Confocal Microscopy

For confocal microscopy, four 40 μm-thick skin FFPE specimens were assessed from each sample group. Free-floating skin specimens were deparaffinized, and heat-induced antigen retrieval was performed. Incubations with the primary (anti-human CLDN1: rabbit polyclonal IgG, ab15098, Abcam; anti-human DSG1: mouse monoclonal IgG, 129204, Novus; anti-human CDSN, rabbit polyclonal IgG, HPA054184, Sigma-Aldrich) and secondary (Alexa FluorTM 488 and 555 goat anti-mouse IgG (H+L), Thermo Fisher Scientific) antibodies were performed on free-floating specimens for 48 h at 4 °C and 2 h at room temperature, respectively. Finally, specimens were placed on slides and mounted with VECTASHIELD^®^ HardSet™ Antifade Mounting Medium with DAPI (Vector Laboratories).

Imaging of immunostained samples was carried out on an Olympus FV3000 confocal system using a 60× oil-immersion lens (NA: 1.42). Acquisition settings (laser power, confocal aperture and gain, detector parameters) were identical for all samples. A series of 1 μm-thick optical sections with 0.5 µm separation in the *Z*-axis were acquired. The overall number of optical sections per sample was 11. No pixels for immunostained dots were saturated. Images for figures were processed using the Adobe Photoshop CS5 software.

Line profiles for the green and red channels were measured and plotted using the FIJI image analysis package. Line regions of interest (ROIs) were defined on the optical plane with the brightest signal in the particular channel, ranging from stratum granulosum to stratum basale (as assessed from the channel containing the DAPI stained nuclei). A single line was measured in each image stack. Normalized line profiles were averaged within the same group (GP, SGR, AGR) and plotted using Microsoft Excel. Due to the variable lengths of the regions of interest, only the first 50 micrometers (starting from stratum granulosum) were compared.

The regularity of the DSG1 and CLDN immunostaining was analyzed by measuring the distance between two adjacent immunostained dots along the cell membrane cross-section (a closed line around a DAPI-stained nucleus in the region of stratum granulosum). In a given skin type, 100 inter-dot distances were measured. Distances are presented as box plots where the box represents the 25th, 50th, and 75th percentiles (Q1, Q2, and Q3, respectively) and whiskers indicate the 1.5 times IQR distance (Q3-Q1). Data points are plotted as grey dots. The mean value is shown as a hollow black square within the box.

### 4.8. Statistical Analyses

Data distribution was analyzed using the Kolmogorov–Smirnov test. For normally distributed data, groups were compared using one-way ANOVA followed by the Sidac post hoc test (SGR vs. GP and AGR vs. GP). When data distribution was not normal, a nonparametric test with Dunn’s post hoc test was applied. Differences between the groups were demonstrated using mean ± 95% confidence intervals (CI). *p* values < 0.05 were considered statistically significant. Data were analyzed using GraphPad Prism v8 (GraphPad Software Inc., San Diego, CA, USA) and SPSS 25 (SPSS package for Windows, Chicago, IL, USA).

## Figures and Tables

**Table 1:** Characteristics of the skin samples from healthy individuals (*n* = 24). Abbreviations: AGR, apocrine gland-rich; F, female; GP, gland-poor; M, male; SD, standard deviance; SGR, sebaceous gland-rich.

Healthy Individuals (*n* = 8)			
Healthy individuals	Sex	Age	Localization
GP1	F	43	upper arm
GP2	F	66	ankle
GP3	F	50	upper arm
GP4	F	52	shin
GP5	M	25	upper arm
GP6	M	50	shin
GP7	M	65	upper arm
GP8	M	67	ankle
Mean age ± SD		52.2 ± 14.1	
Healthy Individuals (*n* = 8)			
Healthy individuals	Sex	Age	Localization
AGR1	M	70	axilla
AGR2	M	43	axilla
AGR3	F	48	axilla
AGR4	F	65	axilla
AGR5	F	60	axilla
AGR6	F	38	axilla
AGR7	F	45	axilla
AGR8	F	41	axilla
Mean age ± SD		51.2 ± 12.0	
Healthy Individuals (*n* = 8)			
Healthy individuals	Sex	Age	Localization
SGR1	M	42	nose
SGR2	M	61	forehead
SGR3	F	62	chin
SGR4	M	66	nose
SGR5	M	38	face
SGR6	F	69	forehead
SGR7	M	68	nose
SGR8	M	67	face
Mean age ± SD		59.1 ± 12.2	

## Data Availability

The data that support the findings of this study are available from the corresponding author upon reasonable request.

## References

[1] Bouslimani A., Porto C., Rath C.M., Wang M., Guo Y., Gonzalez A., Berg-Lyon D., Ackermann G., Christensen G.J.M., Nakatsuji T. (2015). Molecular cartography of the human skin surface in 3D. Proc. Natl. Acad. Sci. USA.

[2] Byrd A.L., Belkaid Y., Segre J.A. (2018). The human skin microbiome. Nat. Rev. Genet..

[3] Belkaid Y., Segre J.A. (2014). Dialogue between skin microbiota and immunity. Science.

[4] Jenei A., Dajnoki Z., Medgyesi B., Gáspár K., Béke G., Kinyó Á., Méhes G., Hendrik Z., Dinya T., Törőcsik D. (2019). Apocrine Gland–Rich Skin Has a Non-Inflammatory IL-17–Related Immune Milieu, that Turns to Inflammatory IL-17–Mediated Disease in Hidradenitis Suppurativa. J. Investig. Dermatol..

[5] Béke G., Dajnoki Z., Kapitány A., Gáspár K., Medgyesi B., Póliska S., Hendrik Z., Péter Z., Törőcsik D., Bíró T. (2018). Immunotopographical Differences of Human Skin. Front. Immunol..

[6] Dajnoki Z., Béke G., Kapitány A., Mócsai G., Gáspár K., Rühl R., Hendrik Z., Juhász I., Zouboulis C.C., Bacsi A. (2017). Sebaceous Gland-Rich Skin Is Characterized by TSLP Expression and Distinct Immune Surveillance Which Is Disturbed in Rosacea. J. Investig. Dermatol..

[7] Borkowski A.W., Gallo R.L. (2011). The Coordinated Response of the Physical and Antimicrobial Peptide Barriers of the Skin. J. Investig. Dermatol..

[8] Dorschner R., Pestonjamasp V.K., Tamakuwala S., Ohtake T., Rudisill J., Nizet V., Agerberth B., Gudmundsson G.H., Gallo R. (2001). Cutaneous Injury Induces the Release of Cathelicidin Anti-Microbial Peptides Active Against Group A Streptococcus. J. Investig. Dermatol..

[9] Schauber J., Gallo R.L. (2007). Expanding the Roles of Antimicrobial Peptides in Skin: Alarming and Arming Keratinocytes. J. Investig. Dermatol..

[10] Aberg K.M., Man M.-Q., Gallo R.L., Ganz T., Crumrine D., Brown B.E., Choi E.-H., Kim D.-K., Schröder J.M., Feingold K.R. (2008). Co-Regulation and Interdependence of the Mammalian Epidermal Permeability and Antimicrobial Barriers. J. Investig. Dermatol..

[11] Egawa G., Kabashima K. (2018). Barrier dysfunction in the skin allergy. Allergol. Int..

[12] Egawa G., Kabashima K. (2016). Multifactorial skin barrier deficiency and atopic dermatitis: Essential topics to prevent the atopic march. J. Allergy Clin. Immunol..

[13] McGovern J., Meinert C., De Veer S., Hollier B., Parker T., Upton Z., De Veer S. (2016). Attenuated kallikrein-related peptidase activity disrupts desquamation and leads to stratum corneum thickening in human skin equivalent models. Br. J. Dermatol..

[14] Kishibe M. (2019). Physiological and pathological roles of kallikrein-related peptidases in the epidermis. J. Dermatol. Sci..

[15] Candi E., Schmidt R., Melino G. (2005). The cornified envelope: A model of cell death in the skin. Nat. Rev. Mol. Cell Biol..

[16] Ishida-Yamamoto A., Kishibe M. (2011). Involvement of corneodesmosome degradation and lamellar granule transportation in the desquamation process. Med. Mol. Morphol..

[17] Grice E.A., Segre J.A. (2011). The Skin Microbiome. Nat. Rev. Microbiol..

[18] Kleesz P., Darlenski R., Fluhr J. (2012). Full-Body Skin Mapping for Six Biophysical Parameters: Baseline Values at 16 Anatomical Sites in 125 Human Subjects. Ski. Pharmacol. Physiol..

[19] Komatsu N., Tsai B., Sidiropoulos M., Saijoh K., Levesque M.A., Takehara K., Diamandis E. (2006). Quantification of Eight Tissue Kallikreins in the Stratum Corneum and Sweat. J. Investig. Dermatol..

[20] Komatsu N., Saijoh K., Toyama T., Ohka R., Otsuki N., Hussack G., Takehara K., Diamandis E. (2005). Multiple tissue kallikrein mRNA and protein expression in normal skin and skin diseases. Br. J. Dermatol..

[21] Stefansson K., Brattsand M., Roosterman D., Kempkes C., Bocheva G., Steinhoff M., Egelrud T. (2008). Activation of Proteinase-Activated Receptor-2 by Human Kallikrein-Related Peptidases. J. Investig. Dermatol..

[22] Nadeau P., Henehan M., De Benedetto A. (2018). Activation of protease-activated receptor 2 leads to impairment of keratinocyte tight junction integrity. J. Allergy Clin. Immunol..

[23] Henehan M., De Benedetto A. (2019). Update on protease-activated receptor 2 in cutaneous barrier, differentiation, tumorigenesis and pigmentation, and its role in related dermatologic diseases. Exp. Dermatol..

[24] Koziel J., Potempa J. (2012). Protease-armed bacteria in the skin. Cell Tissue Res..

[25] Li W., Zhang Z.-W., Luo Y., Liang N., Pi X.-X., Fan Y.-M. (2020). Molecular epidemiology, in vitro susceptibility and exoenzyme screening of Malassezia clinical isolates. J. Med. Microbiol..

[26] White T., Findley K., Dawson T., Scheynius A., Boekhout T., Cuomo C., Xu J., Saunders C.W. (2014). Fungi on the Skin: Dermatophytes and Malassezia. Cold Spring Harb. Perspect. Med..

[27] Nguyen T.T.H., Myrold D.D., Mueller R.S. (2019). Distributions of Extracellular Peptidases Across Prokaryotic Genomes Reflect Phylogeny and Habitat. Front. Microbiol..

[28] Dessinioti C., Katsambas A.D. (2010). The role of Propionibacterium acnes in acne pathogenesis: Facts and controversies. Clin. Dermatol..

[29] Williams M.R., Nakatsuji T., Sanford J., Vrbanac A.F., Gallo R.L. (2016). Staphylococcus aureus Induces Increased Serine Protease Activity in Keratinocytes. J. Investig. Dermatol..

[30] Hirasawa Y., Takai T., Nakamura T., Mitsuishi K., Gunawan H., Suto H., Ogawa T., Wang X.-L., Ikeda S., Okumura K. (2009). Staphylococcus aureus Extracellular Protease Causes Epidermal Barrier Dysfunction. J. Investig. Dermatol..

[31] Ujiie I., Ujiie H., Iwata H., Shimizu H. (2019). Clinical and immunological features of pemphigus relapse. Br. J. Dermatol..

[32] Schmidt E., Kasperkiewicz M., Joly P. (2019). Pemphigus. Lancet.

[33] Kasperkiewicz M., Ellebrecht C.T., Takahashi H., Yamagami J., Zillikens D., Payne A.S., Amagai M. (2017). Pemphigus. Nat. Rev. Dis. Primers.

[34] Savignac M., Simon M., Edir A., Guibbal L., Hovnanian A. (2014). SERCA2 Dysfunction in Darier Disease Causes Endoplasmic Reticulum Stress and Impaired Cell-to-Cell Adhesion Strength: Rescue by Miglustat. J. Investig. Dermatol..

[35] Dhitavat J., Fairclough R., Hovnanian A., Burge S. (2004). Calcium pumps and keratinocytes: Lessons from Darier’s disease and Hailey-Hailey disease. Br. J. Dermatol..

[36] Sudbrak R., Brown J.M., Dobson-Stone C., Carter S., Ramser J., White J., Healy E., Dissanayake M., Larrègue M., Perrussel M. (2000). Hailey-Hailey disease is caused by mutations in ATP2C1 encoding a novel Ca^2+^ pump. Hum. Mol. Genet..

[37] Savignac M., Edir A., Simon M., Hovnanian A. (2011). Darier disease: A disease model of impaired calcium homeostasis in the skin. Biochim. Biophys. Acta (BBA) Bioenerg..

